# Vitamin D and MAFLD: a bibliometric and visual analysis from 2007 to 2024

**DOI:** 10.3389/fnut.2025.1558026

**Published:** 2025-06-06

**Authors:** Jiaguo Zhang, Sipeng Wu, Junqian Huang, Xuehua Sun, Jianjie Chen, Binyi Shang

**Affiliations:** ^1^Department of Hepatology, Shuguang Hospital, Shanghai University of Traditional Chinese Medicine, Shanghai, China; ^2^Chen Jianjie National Famous Elderly Chinese Medicine Experts Inheritance Workshop, Shanghai, China

**Keywords:** metabolic-associated fatty liver disease, vitamin D, insulin resistance, obesity, osteoporosis, VOSviewer, CiteSpace, visualization and analysis

## Abstract

**Background:**

Vitamin D plays a critical role in the mechanism of metabolic fatty liver disease. Emerging evidence suggests its potential as both a diagnostic biomarker and therapeutic target. Despite growing research interest, systematic analyses of this field remain limited.

**Purpose:**

This study conducts a bibliometric and visual analysis of literature on the link between vitamin D and metabolism-related fatty liver disease, mapping the research landscape, trends, hotspots, and frontiers to inform future investigations.

**Methods:**

The Web of Science Core Collection (WoSCC) database was comprehensively explored for literature pertaining to metabolism-related fatty liver disease and vitamin D from 2007 to 2024. Using CiteSpace (v6.1. R6) and VOSviewer (v1.6.20) software, we evaluated publication trends, regional and national contributions, institutional outputs, journal allocation, author collaborations, reference citations, and keyword patterns.

**Results:**

A total of 480 publications were analyzed, demonstrating a consistent annual increase. China, the United States, and Italy emerged as leading contributors, with Shanghai Jiao Tong University, Capital Medical University, and Sichuan University being the most productive institutions. The most cited and prolific author was Ilaria Barchetta. *Hepatology* ranked as the journal with the highest volume of publications in this field. The top most frequently used keywords are “vitamin D,” “NAFLD,” and “insulin resistance.” Burst detection indicated “T2DM” and “acid” as emerging research foci.

**Conclusion:**

This study provides insights into current trends and prospects in research on vitamin D and metabolism-associated fatty liver disease, focusing on insulin resistance, obesity, osteoporosis, and ACID. While the efficacy of vitamin D supplementation remains uncertain, the findings offer guidance for broader future studies.

## Introduction

Metabolic-associated fatty liver disease (MAFLD) is the latest official name for non-alcoholic fatty liver disease (NAFLD). The diagnostic framework for MAFLD is founded on histological (liver biopsy), imaging, and blood biomarker evidence of hepatic steatosis (fatty degeneration of hepatocytes), in combination with one of the following three criteria: overweight/obesity, diabetes mellitus (type 2 diabetes mellitus), and metabolic dysfunction. Exclusion of other liver diseases, including alcoholic, autoimmune, or viral hepatitis (1). MAFLD is now a growing disease in the world, affecting more than 25% of the world’s population (2).

Vitamin D (VD) is a lipid-soluble vitamin derived from sterol compounds. Numerous studies have shown that VD may be involved in the immune-inflammatory response and that it may have a role in modulating autophagy, reducing leukocyte differentiation and activation, and decreasing oxidative stress (3, 4). VD is also involved in insulin resistance, which affects the pathogenesis of MAFLD. It has been documented that VD increases the insulin sensitivity of cells (5). The prevalence of vitamin D deficiency has been reported to increase in parallel with the prevalence of obesity (6). Although the underlying mechanisms by which VD is associated with MAFLD are not fully understood at this time, it has been demonstrated in the literature that VD deficiency is prevalent in subjects with MAFLD (7). Therefore, VD is an important factor in the future diagnosis and treatment of MAFLD and may become an important indicator for the diagnosis of MAFLD.

CiteSpace and VOSviewer are bibliometric and visualization tools that enable the visual analysis of scientific literature in a specific field. They play a crucial role in identifying research frontiers and guiding the selection of research directions (8, 9). There is a relative scarcity of bibliometric studies focusing on the intersection of vitamin D and MAFLD. Consequently, we selected pertinent literature from the Web of Science database and systematically collected data regarding authors, institutions, countries, journals, citations, and keywords. These tools are used to identify keyword bursts, investigate research hotspots and emerging frontiers within the field, and offer valuable insights for future studies examining the relationship between vitamin D and MAFLD.

## Materials and methods

### Data sources and analyses

**Figure 1:**
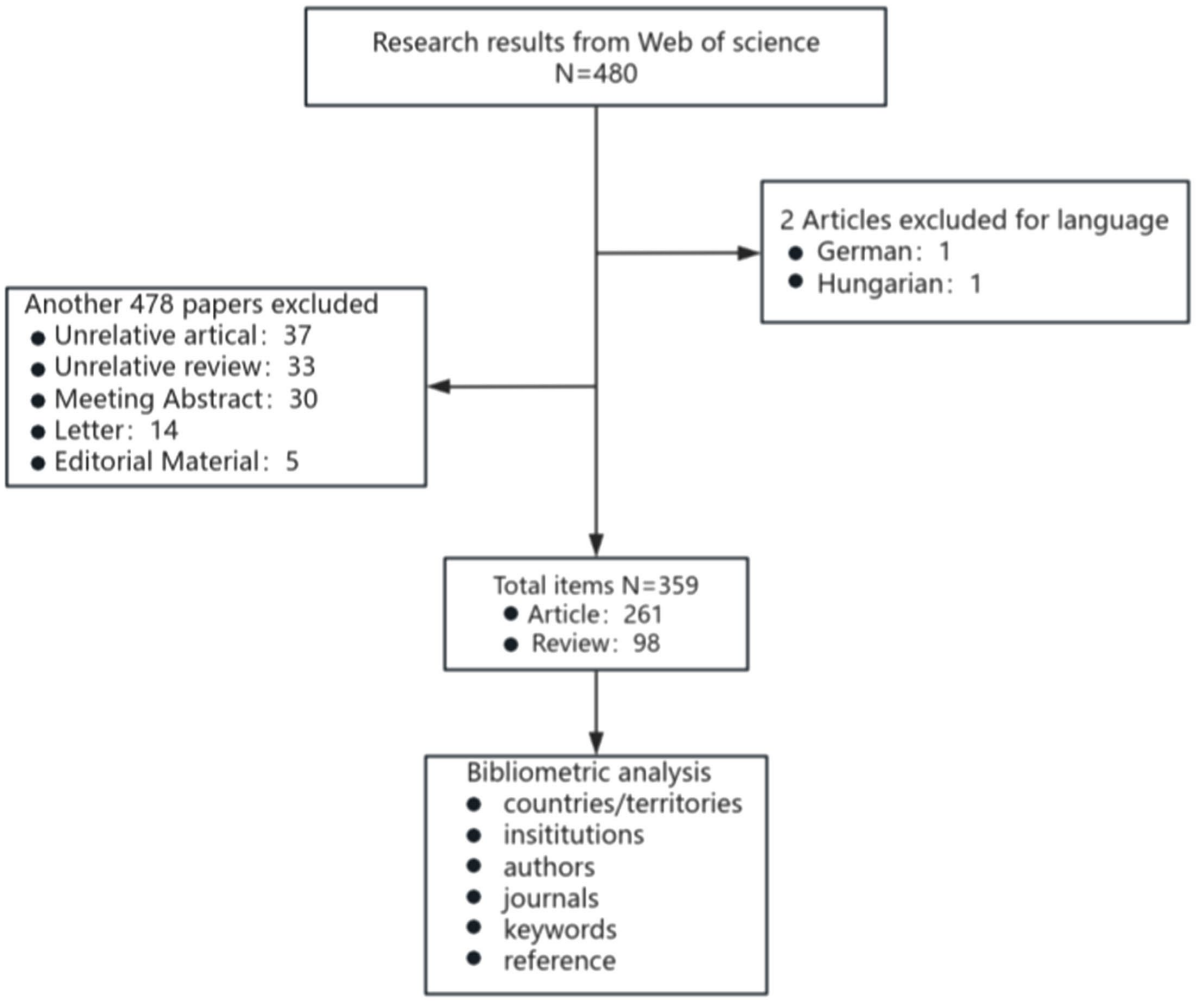
Flow chart of the retrieval process in this study.

The Web of Science Core Collection (WoSCC, Clarivate) on 1 October 2024 was screened for literature between 1 January 2007 and 1 October 2024. The search method was TS = (“nonalcoholic fatty liver disease” OR “NAFLD” OR “non-alcoholic fatty liver” OR “metabolic dysfunction-associated fatty liver disease” OR “MAFLD”) AND TS = (“vitamin d” OR “dihydroxyvitamin d”). After searching according to the above methods, 480 documents were identified. Based on the specific requirements of this study, we established a set of clear inclusion criteria: Papers meeting the analysis criteria must meet the following conditions: (1) be written in English, (2) be limited to original research and review articles, and (3) focus on MAFLD with involvement of VD. Using this strategy, we identified 359 publications, comprising 261 articles and 98 reviews (The specific flow chart is shown in Figure 1). All records, encompassing authors, titles, abstracts, keywords, and cited literature, were systematically downloaded and subsequently imported into VOSviewer (v1.6.20) and CiteSpace (v6.1. R6) for comprehensive analysis. The software came with a duplicate check function, and no duplicate literature was found. Keywords with similar meanings were consolidated into the exported data.

## Result

### Publication output and temporal trend

**Figure 2:**
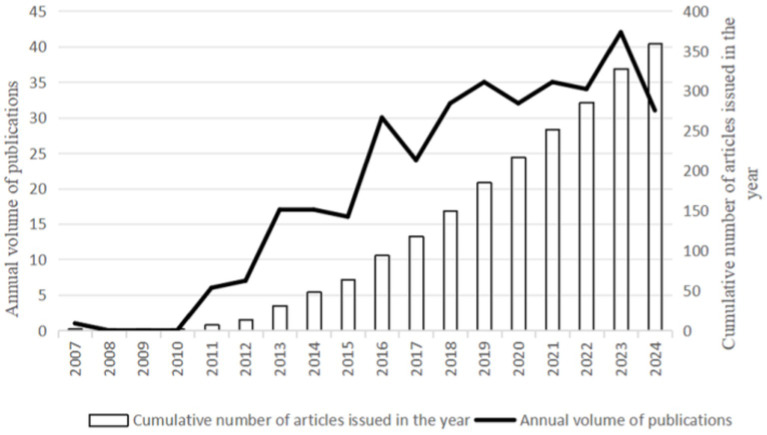
Overall trend of published papers over the years.

From 2007 to 2024, WoSCC retrieved 359 literature, including 261 articles and 98 reviews, for further data extraction. Figure 2 shows the number of published literature in the last 20 years, with no more than 30 papers on VD with MAFLD from 2007 to 2012. However, from 2013 onwards, the number of publications steadily increased, ultimately reaching a peak in annual publications in 2023 (*n* = 42). The WOS citation report indicates that these documents have been cited 17,858 times, with an average of 37.42 citations per document. These data indicate that the field of vitamin D and MAFLD has garnered extensive attention from various stakeholders, including academic researchers, healthcare professionals, and public health organizations.

### Distribution of authors and co-cited authors

From Table 1, it can be seen that Barchetta, Ilaria, from Sapienza University of Rome, Italy, published the most papers, eight publications. Following closely are Angelico, Francesco, also from Sapienza University of Rome, Italy (*n* = 5), and Zhang, Wei, from Shanghai Jiao Tong University, China (*n* = 4). Four of the top 10 authors are from the Sapienza University of Rome, which demonstrates the high level of interest in the field of VD and MAFLD at these institutions.

**Table 1:** Top 10 authors studying vitamin D and MAFLD.

Rank	Author	Count	Country	Institution
1	Barchetta, I	8	Italy	Sapienza University of Rome
2	Angelico, F	5	Italy	Sapienza University Rome
3	Zhang, W	4	China	Shanghai Jiao Tong University
4	Cavallo, M. G	4	Italy	Sapienza University Rome
5	Baik, S. H	4	South Korea	Korea University
6	Gerken, G	3	Germany	University of Duisburg Essen
7	Canbay, A	3	Germany	Ruhr University Bochum
8	Byrne, C. D	3	England	Univ Hosp Southampton
9	Bertoccini, L	3	Italy	Sapienza University Rome
10	Bechmann, L. P	3	Germany	Ruhr University Bochum

From the 10 most cited authors in Table 2, we observe that the top two authors, Barchetta, Ilaria (*n* = 198) and Targher G (*n* = 160), are from Italy, and we also find that Barchetta, Ilaria, is both the author with the maximum number of publications and the most referenced author. Meanwhile, seven authors are from the United States. This indicates that the field has received extensive attention from relevant researchers in the United States and Italy, and the authors with the top 10 citation frequency rankings have made important contributions to the field of VD and MAFLD and attained internationally recognized scholarly influence.

**Table 2:** Top 10 cited authors on vitamin D and MAFLD.

Rank	Cited author	Cited frequency	Country	Institution
1	Barchetta, Ilaria	198	Italy	Sapienza University of Rome
2	Targher G	160	Italy	IRCCS Sacro Cuore Don Calabria Hospital
3	Eliades M	130	USA	University of Maryland, Baltimore
4	Younossi ZM	94	USA	Inova Fairfax Hospital
5	Holick MF	94	USA	Boston University
6	Roth CL	89	USA	Seattle Children’s Hospital
7	Chalasani N	84	USA	University of South Florida
8	Sharif N	70	Iran	Kashan Univ Med Sci
9	Dasarathy J	59	USA	MetroHealth System
10	Angulo P	58	USA	University of Kentucky

**Figure 3:**
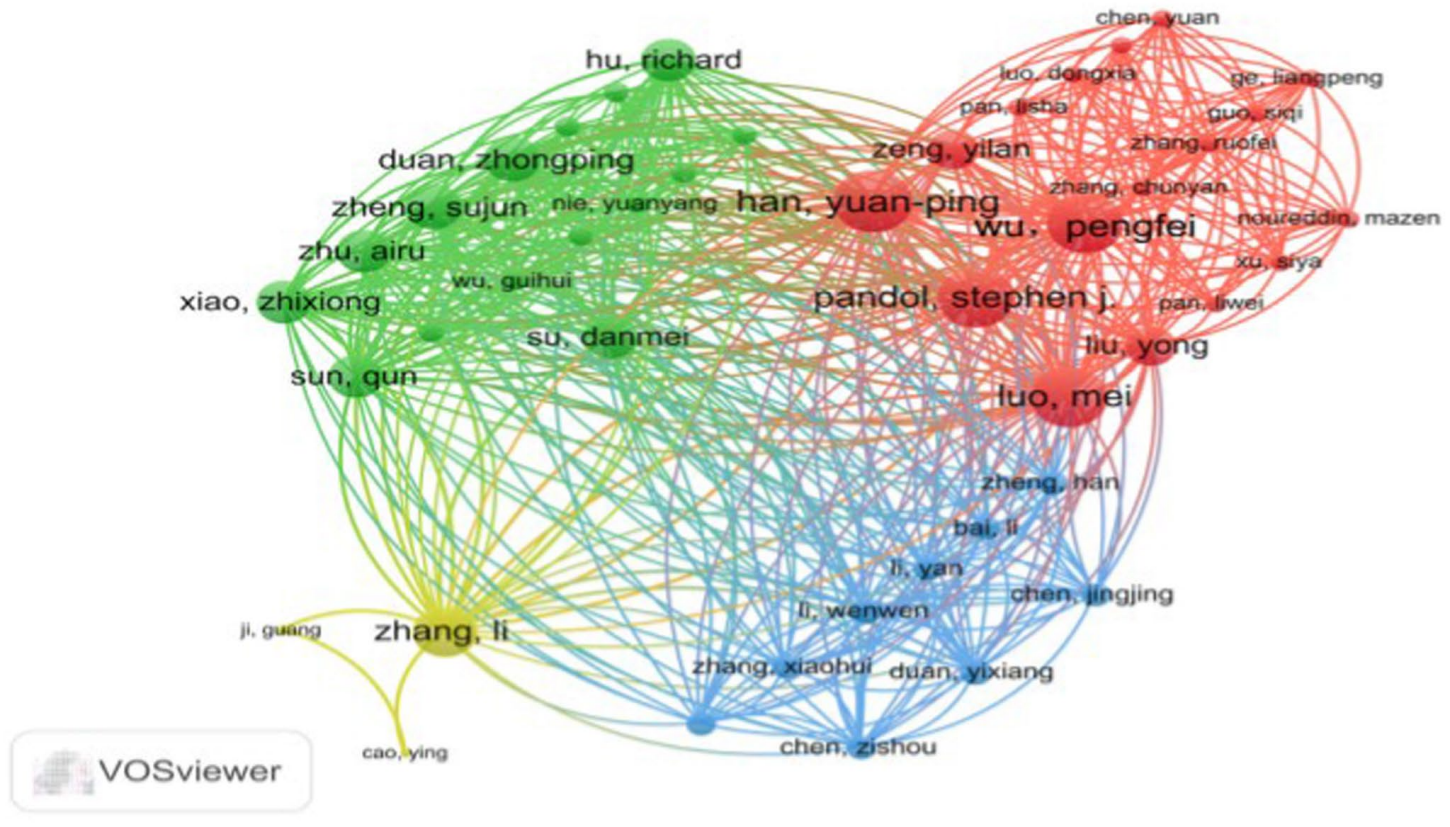
Author partnership network.

Figure 3, generated by VOSviewer, utilizes distinct colors to symbolize closely connected clusters. A visual examination of the author collaboration network reveals that in the red cluster, Wu, Pengfei, collaborates closely with Han, Yuanping, Luo, Mei, and Pandol, Stephen J, among others. The yellow cluster forms a collaboration network centered around Zhang, Li, with participation from Ji, Guang and Cao, and Ying. The green cluster features a collaborative group consisting of Su, Danmei, Xiao, Zhixiong, Sun, Qun, Zhu, Airu, and Duan, Zhongping. Finally, the blue cluster represents a collaborative network built by Chen, Jingjing, Duan, Yixiang, and Cheng, Zisou among others.

**Figure 4:**
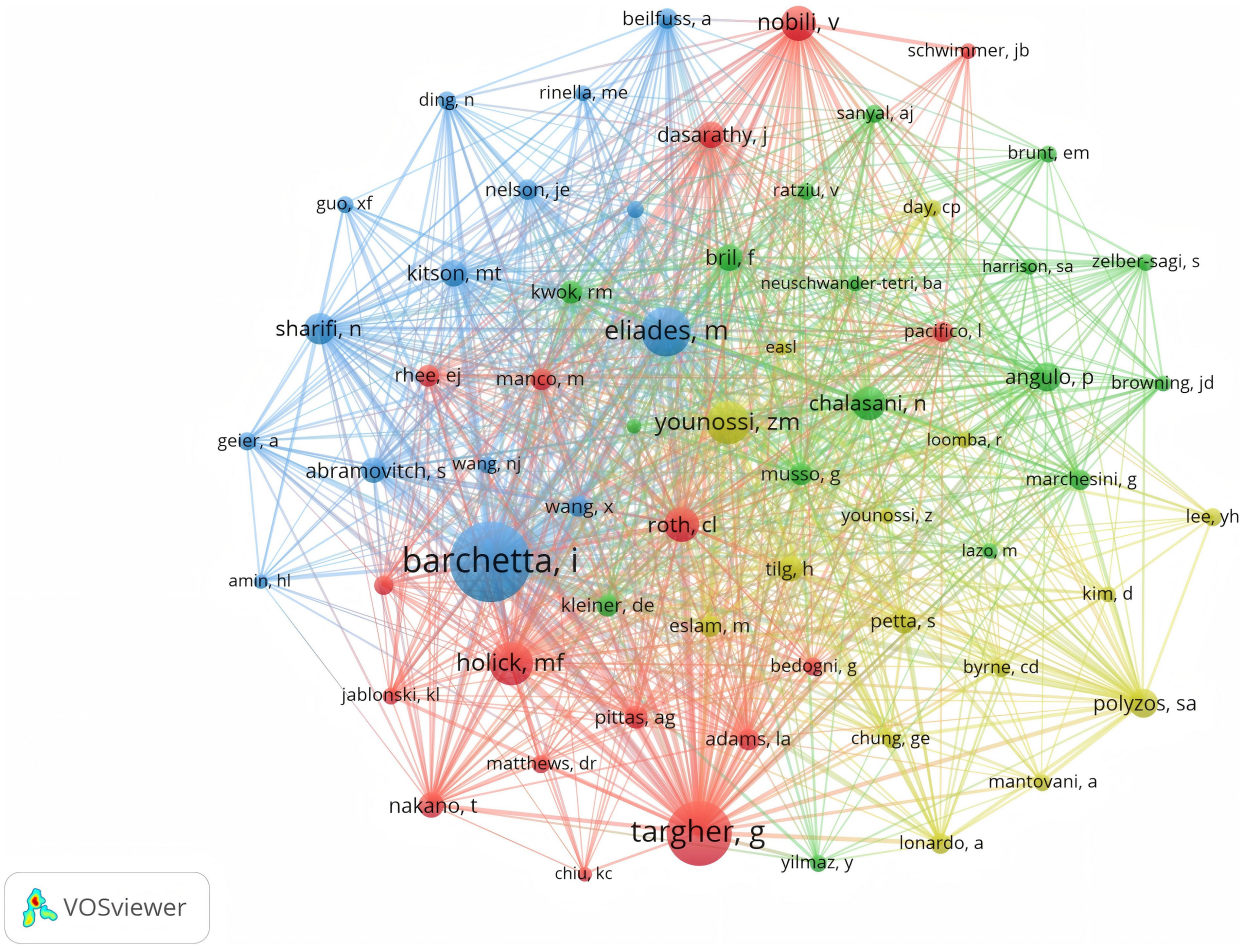
Co-cited author relationship network.

As illustrated in Figure 4, Barchetta Ilaria holds the highest number of co-citations, followed by Targher G and Eliades M, each with over 150 co-citations. In addition, these three authors are also the most frequently cited researchers in the field. In summary, they hold a very high academic status in this field.

### Distribution of countries/areas and institutions

**Figure 5:**
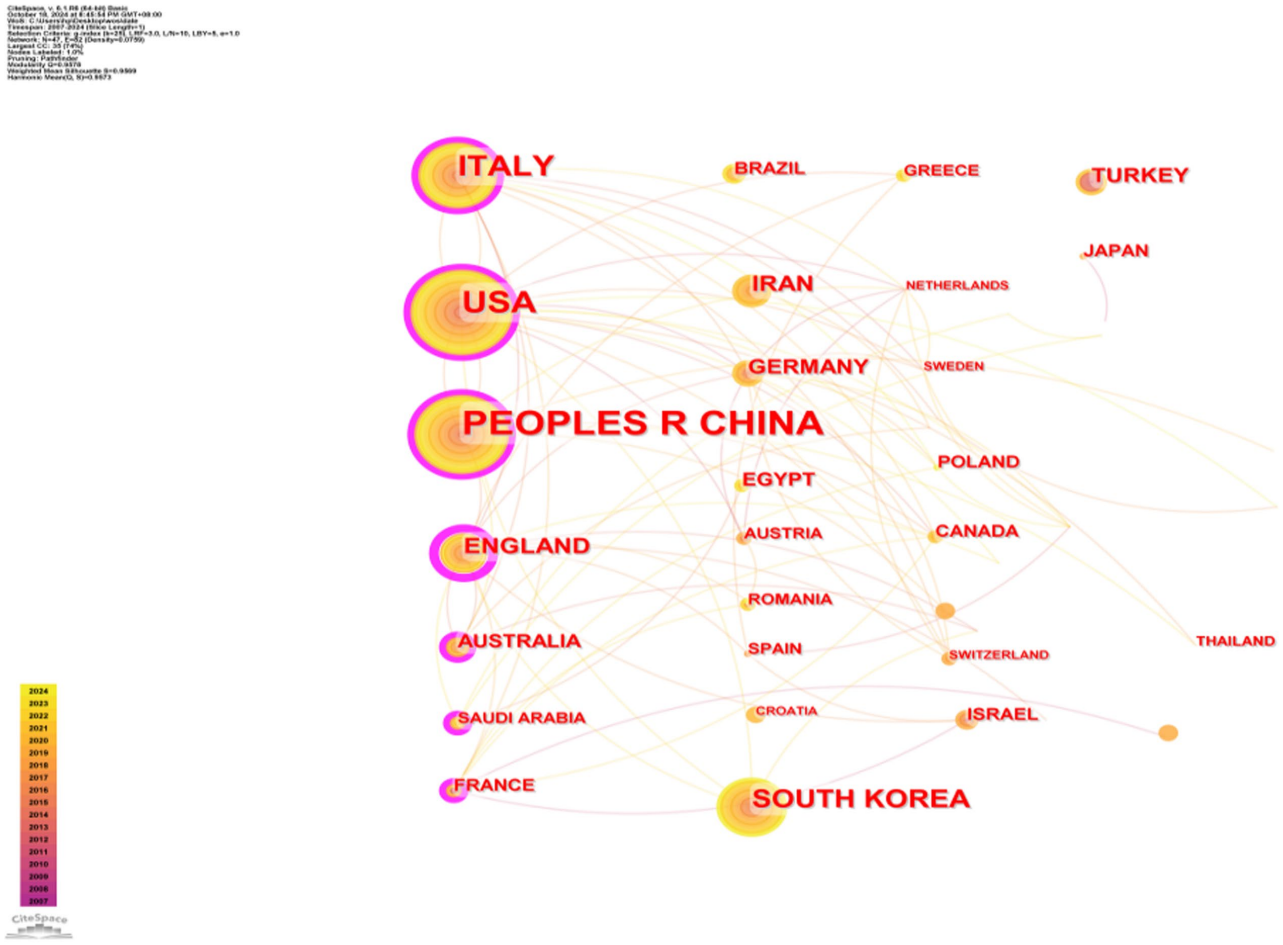
Visualization map of countries.

**Figure 6:**
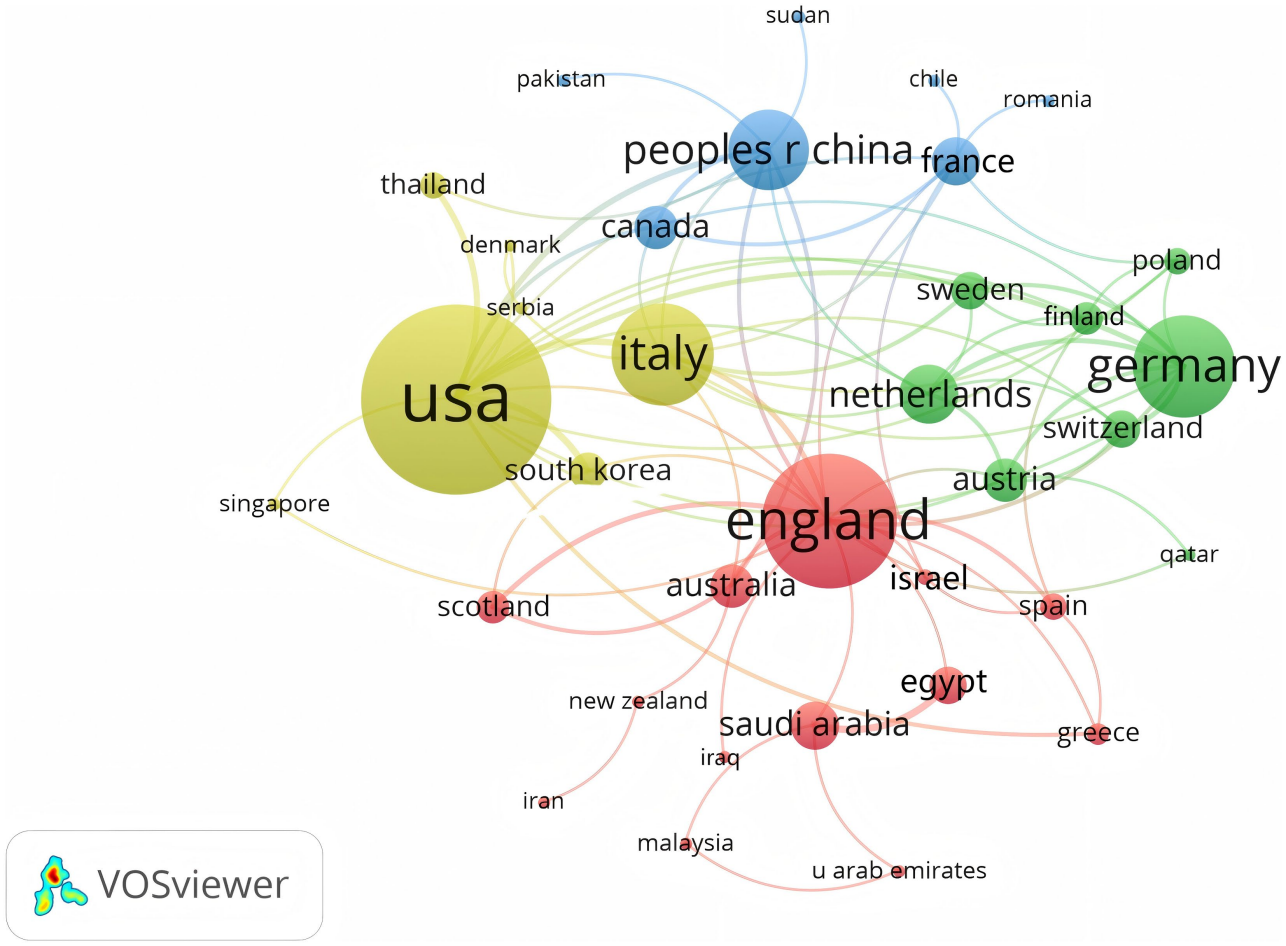
National partnership networks.

A total of 350 organizations in 47 countries/regions published relevant papers on VD and MAFLD. As shown in Table 3, China had the greatest volume of publications (*n* = 92), followed by the United States (*n* = 55), Italy (*n* = 49), South Korea (*n* = 29), the United Kingdom (*n* = 20), Iran (*n* = 19), Germany (*n* = 13), Turkey (*n* = 13), Australia (*n* = 12), and Egypt (*n* = 12). The top 3 of the top 10 organizations are all from China, with Shanghai Jiao Tong University (*n* = 7) topping the list. These data show that China has a prominent contribution to the field of VD and MAFLD and has extensive research in this area. VOSviewer provides valuable insights into influential researchers and potential collaborators, aiding in the establishment of research partnerships (10). In Figure 5, we can see that the nodes of China, USA, Italy, UK, Australia, France, and Saudi Arabia are marked by purple circles (11, 12). This figure utilizes the betweenness centrality metric to evaluate the significance of each node within the network. Nodes with a centrality value greater than 0.1 are highlighted with purple circles, indicating that these countries play a pivotal role in shaping the research landscape in this field. As depicted in Figure 6, the different colors represent clusters of close connections, categorizing countries with strong cooperation into four distinct groups. Green part: Germany has closer ties with Sweden, the Netherlands, Austria, Poland, etc. Yellow part: The United States has close ties with Italy, South Korea, Singapore, etc. Blue part: China has strong ties with Canada, France, Sudan, etc. Red section: The UK has more links with Australia, Israel, Saudi Arabia, and Scotland.

**Table 3:** Top 10 countries and institutions on VD and MAFLD.

Rank	Publications	Country	Publications	Institution	Original country
1	92	Peoples R China	7	Shanghai Jiao Tong Univ	Peoples R China
2	55	USA	6	Capital Med Univ	Peoples R China
3	49	Italy	4	Sichuan Univ	Peoples R China
4	29	South Korea	4	Med Univ Vienna	Austria
5	20	England	4	Cleveland Clin	USA
6	19	Iran	4	Cedars Sinai Med Ctr	USA
7	13	Germany	4	Catholic Univ Korea	South Korea
9	12	Australia	4	Azienda Osped Univ Integrata Verona	Italy
10	12	Egypt	4	Aristotle Univ Thessaloniki	Greece

**Figure 7:**
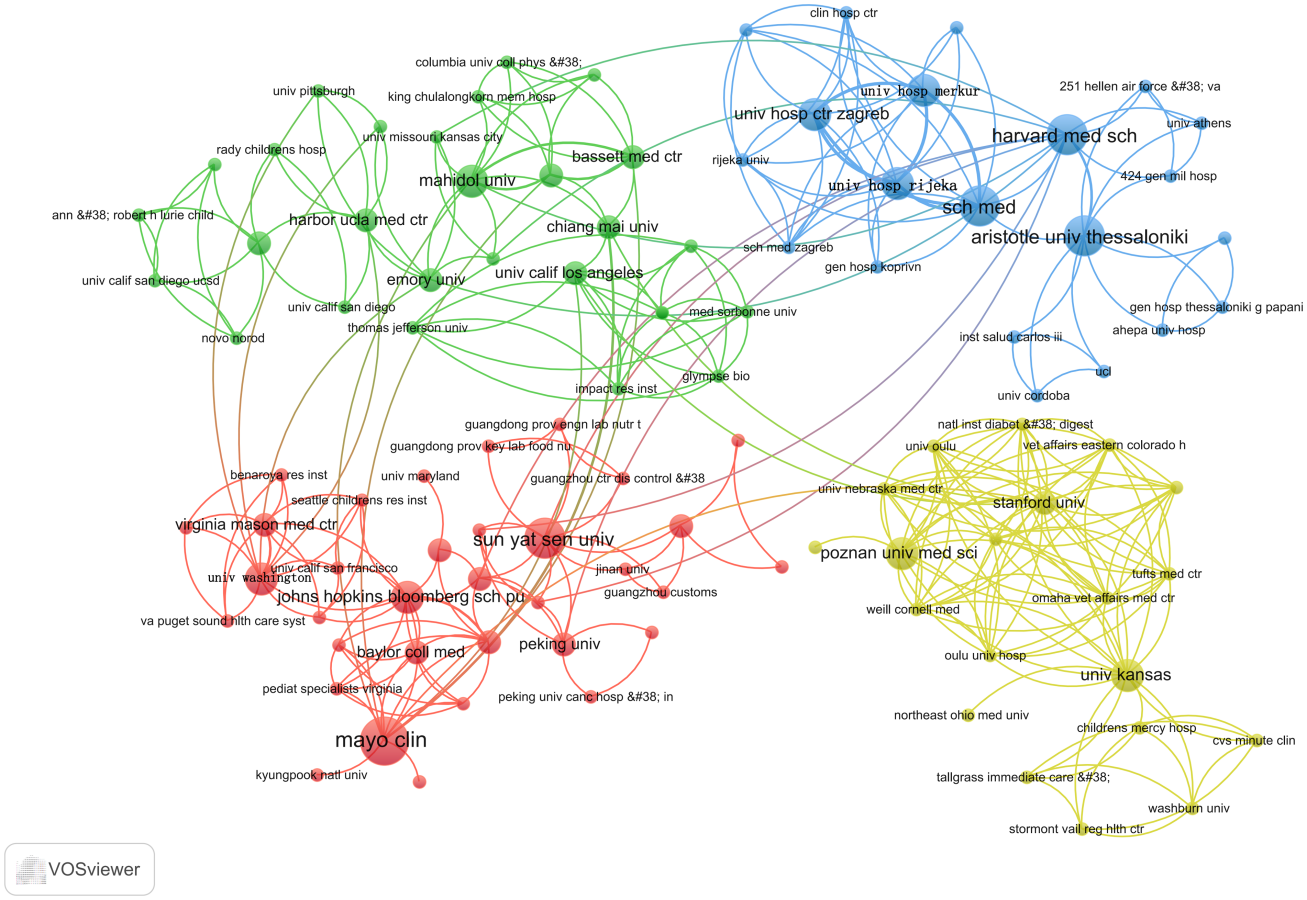
Network of institutional partnerships.

Figure 7 shows that the green section forms a network comprising Mahidol University, Chiang Mai University, University of California, Los Angeles, and Harbor UCLA Medical Center, among others; the red part is a cluster of relationships formed by sun Yat-sen University, the University of Washington, the Johns Hopkins Bloomberg School of Public Health, and Mayo Clinic, among others, forming clusters of relationships. The yellow part is the relational grid formed by Stanford University, Poznan University of Medical Sciences, and the University of Kansas. The blue part is the collaborative network formed by sch med, Harvard Medical School, and Aristotle University of Thessaloniki.

### Journal distribution

Table 4 shows that the highest citation frequency was in hepatology (*n* = 309), followed by J Hepatol (*n* = 275), PLOS One (*n* = 203), and Gastroenterology (*n* = 660). Second, the JIF of a journal is an important reference factor in assessing journals and included literature. Among the top 10 journals, the highest JIF was found in gastroenterology (IF = 29.4), followed by J Hepatol (IF = 26.8) and hepatology (IF = 13.5). Journal citation reports classify journals in the same WoS category as Q1–Q4 based on their JIF values, according to the order of their ranking. As shown in Table 4, almost all journals are classified as Q1. These results indicate that the aforementioned journals provide scholars who wish to explore the relationship between VD and MAFLD with clear directions for literature retrieval and offer highly valuable references for related research.

**Table 4:** Top 10 journals in order of publications and citations.

Rank	Cited journal	Total citations	JIF (JCR 2024)	JCR quartile
1	Hepatology	309	13.5	Q1
2	J Hepatol	275	26.8	Q1
3	PLOS One	203	2.9	Q1
4	Gastroenterology	203	29.4	Q1
5	J Clin Endocr Metab	196	5.0	Q1
6	Nutrients	185	5.9	Q1
7	World J Gastroentero	184	4.3	Q2
8	Aliment Pharmther	176	6.6	Q1
9	AM J Clin Nutr	168	8	Q1
10	Liver INT	159	6.7	Q1

### Highly cited reference analysis

As illustrated in Table 5, *Meta-analysis: vitamin D and non-alcoholic fatty liver disease* by Eliades et al. (7) was the most cited (*n* = 46). Through a systematic review and meta-analysis of approximately 5,000 NAFLD cases and 8,000 controls, this study found that VD levels in NAFLD subjects were lower compared to the control group, confirming that VD deficiency is prevalent among NAFLD subjects. This observation indicates that vitamin D’s anti-inflammatory and immunomodulatory properties may serve as a crucial mechanism underlying its influence on the progression and severity of NAFLD. However, the directionality of these results remains uncertain (7).

**Table 5:** Top 10 cited references of explosive intensity.

Rank	Citations	Title	Author	Journal	Year	DOI
1	46	Meta-analysis: vitamin D and non-alcoholic fatty liver disease	Eliades M	Aliment Pharm Ther	2013	10.1111/apt.12377
2	40	Strong association between non-alcoholic fatty liver disease (NAFLD) and low 25(OH) vitamin D levels in an adult population with normal serum liver enzymes	Barchetta I	BMC Med	2011	10.1186/1741-7015-9-85
3	40	Relationship of vitamin D with insulin resistance and disease severity in non-alcoholic steatohepatitis	Bril F	J Hepatol	2015	10.1016/j.jhep.2014.08.040
4	38	Vitamin D deficiency in obese rats exacerbates non-alcoholic fatty liver disease and increases hepatic resistin and Toll-like receptor activation	Roth CL	Hepatology	2012	10.1002/hep.24737
5	38	No effects of oral vitamin D supplementation on non-alcoholic fatty liver disease in patients with type 2 diabetes: a randomized, double-blind, placebo-controlled trial	Barchetta I	BMC Med	2016	10.1186/s12916-016-0638-y
6	36	Vitamin D and Metabolic Dysfunction-associated fatty liver disease (MAFLD): An Update	Barchetta I	Nutrients	2020	10.3390/nu12113302
7	35	Vitamin D: a new player in non-alcoholic fatty liver disease?	Eliades M	World J Gastroentero	2015	10.3748/wjg.v21.i6.1718
8	34	Does vitamin D improve liver enzymes, oxidative stress, and inflammatory biomarkers in adults with non-alcoholic fatty liver disease? A randomized clinical trial	Sharifi N	Endocrine	2014	10.1007/s12020-014-0336-5
9	31	Global epidemiology of non-alcoholic fatty liver disease: Meta-analytic assessment of prevalence, incidence, and outcomes	Younossi ZM	Hepatology	2016	10.1002/hep.28431
10	29	Liver vitamin D receptor, CYP2R1, and CYP27A1 expression: relationship with liver histology and vitamin D3 levels in patients with non-alcoholic steatohepatitis or hepatitis C virus	Barchetta I	Hepatology	2012	10.1002/hep.25930

The article with the second highest number of citations is *Strong association between non-alcoholic fatty liver disease (NAFLD) and low 25(OH) vitamin D levels in an adult population with normal serum liver enzymes* (*n* = 40). This study revealed a strong independent correlation between low 25(OH) vitamin D levels and NAFLD. The analysis revealed an inverse relationship between serum 25(OH) vitamin D levels and the severity of NAFLD, indicating that vitamin D may exert a dose-dependent effect on hepatic fat accumulation (13).

However, Bril et al. (14) published *Relationship of vitamin D with insulin resistance and disease severity in non-alcoholic steatohepatitis* (*n* = 40), showed that low plasma VD was associated with liver fat accumulation or severity of NASH, and no significant association was found between plasma VD levels and insulin resistance.

This clinical trial grouped 239 subjects according to their metabolic parameters (BMI, total adiposity, prevalence of T2DM, and glycated hemoglobin) and demonstrated that plasma VD concentrations did not correlate with hepatic triglyceride content, did not significantly correlate with insulin sensitivity, and did not differ from the severity of hepatic histology in patients with NASH, respectively (14).

### Keyword analysis

#### Keyword co-occurrence

Keywords encapsulate the core content of an article serving as essential tools for uncovering and analyzing the knowledge frontiers within a given field. From Table 6 we can find that excluding vitamin D (*n* = 223) and NAFLD (*n* = 199) insulin resistance (*n* = 148) metabolic syndrome (*n* = 90) vitamin D deficiency (*n* = 85) and association (*n* = 63) appeared with frequencies that were higher. The keywords with high centrality were hepatic steatosis oxidative stress vitamin D deficiency risk and expression.

**Table 6:** Top 20 keywords about VD and MAFLD.

Rank	Keywords	Count	Centrality	Rank	Keywords	Count	Centrality
1	Vitamin D	223	0.02	11	Hepatic steatosis	45	0.13
2	NAFLD	199	0.04	12	Oxidative stress	41	0.12
3	Insulin resistance	148	0.09	13	Fatty liver disease	38	0.11
4	Metabolic syndrome	90	0.11	14	Fibrosis	36	0.07
5	Vitamin D deficiency	85	0.1	15	Expression	35	0.1
6	Association	63	0.13	16	Vitamin d level	30	0.11
7	Steatohepatitis	58	0.06	17	D supplementation	27	0.08
8	Risk	56	0.1	18	Disease	27	0.07
9	Prevalence	53	0.08	19	Risk factor	25	0.07
10	Obesity	45	0.04	20	Meta-analysis	24	0.05

**Figure 8:**
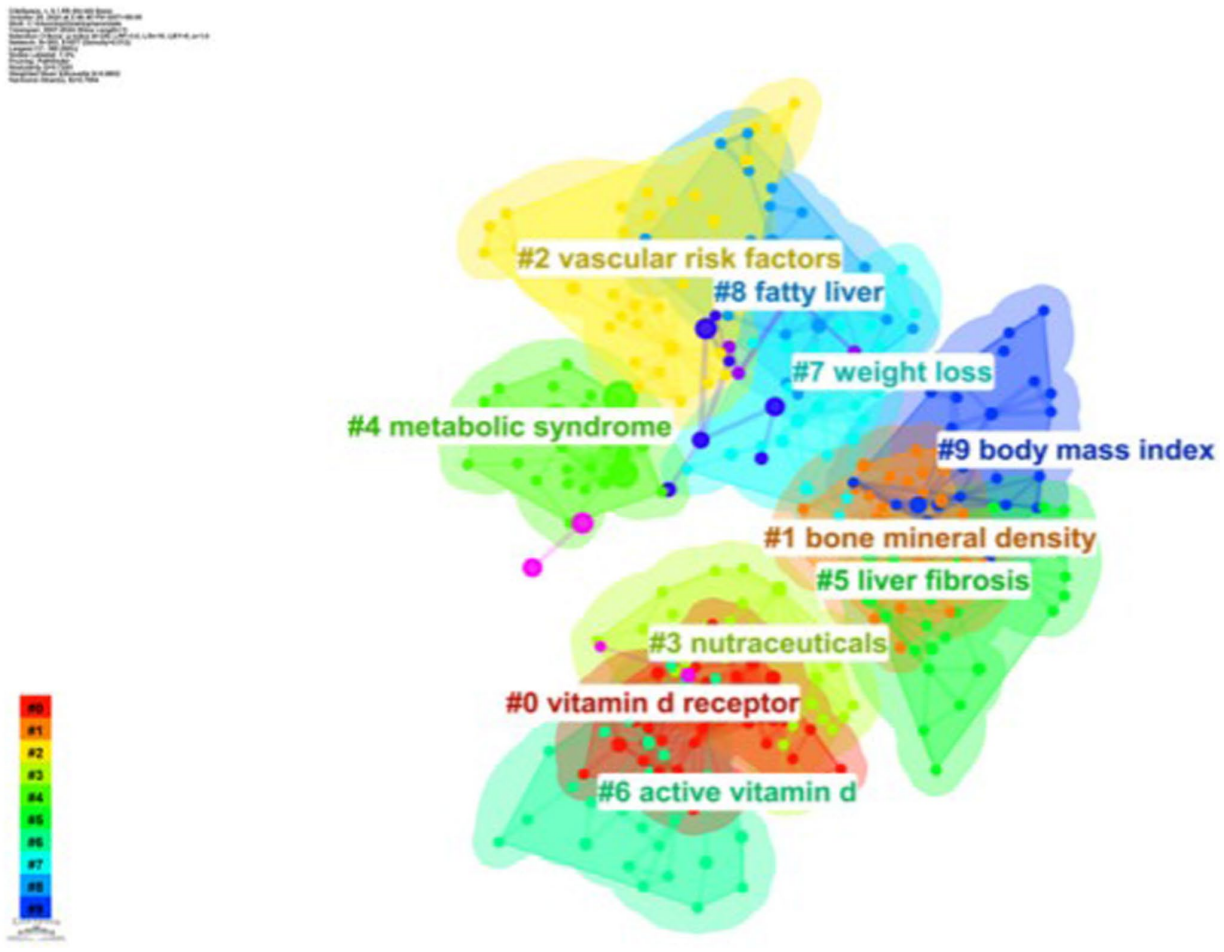
Keyword clustering network.

As shown in Figure 8, based on the co-occurrence of keywords in CiteSpace, which can be categorized into 10 major clusters, research in this area has focused on vitamin D receptor, bone mineral density, vascular risk factors, nutraceuticals, metabolic syndrome, liver fibrosis, active vitamin D, weight loss, fatty liver, and body mass index.

**Figure 9:**
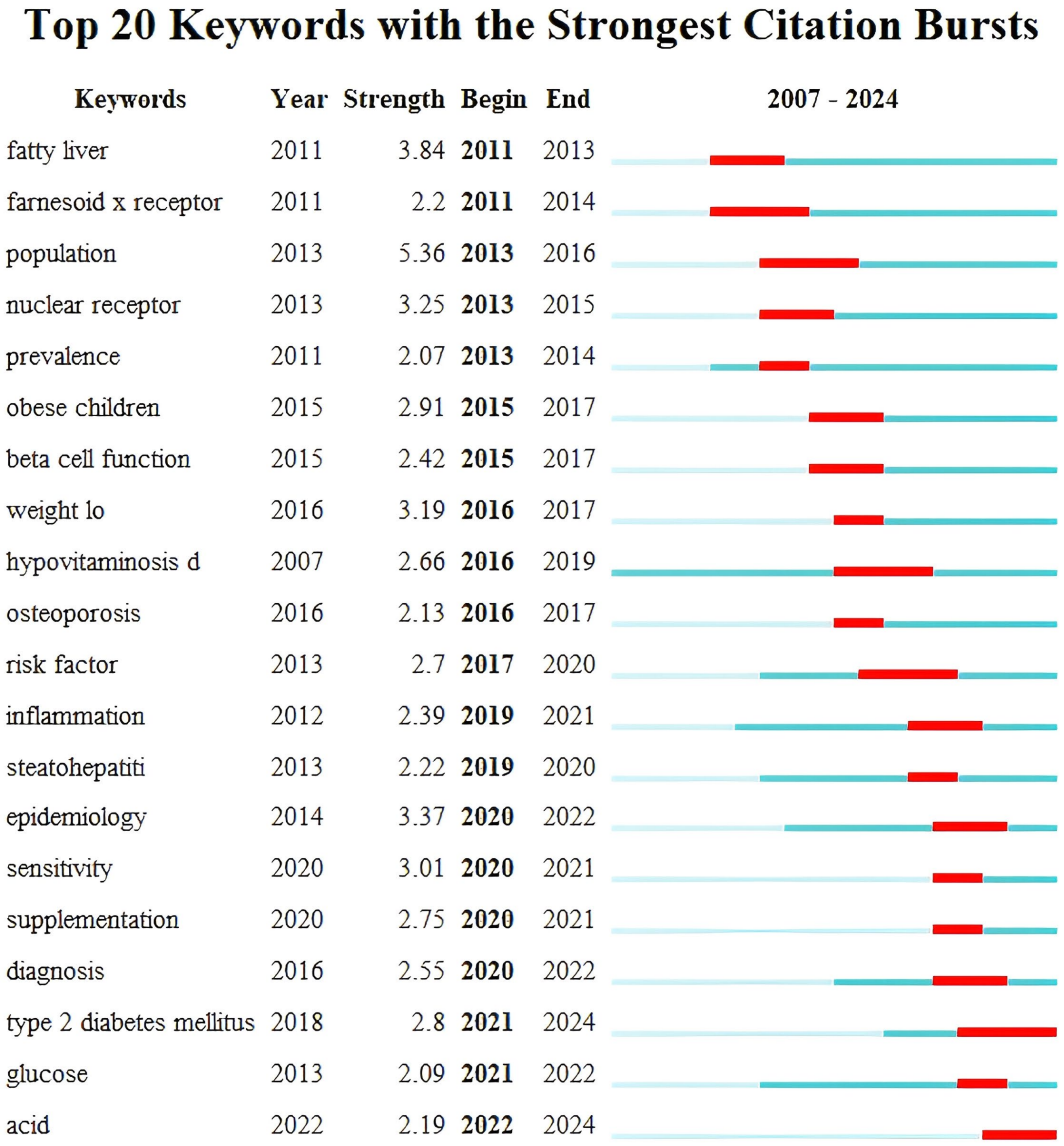
Outbreak time of co-cited documents.

Keyword burst detection was performed using CiteSpace software and in Figure 9 the blue bar corresponds to the time and the red bar indicates the period when the keyword burst. Keyword bursts can mirror emerging academic trends and topics predict the direction of cutting-edge research and identify potential hotspots in the field. The top 20 keyword bursts are listed in chronological order with the earliest burst occurring in 2011 and the latest in 2023. The term “population” has the strongest burst intensity (*n* = 5.36) followed by “fatty liver” and “epidemiology.” The longest-used terms are “hypovitaminosis D,” while “sensitivity” and “supplementation” have been used for a shorter period. Currently “T2DM” and “acid” are still in their burst phase indicating that these topics represent the current research frontiers in this field.

**Figure 10:**
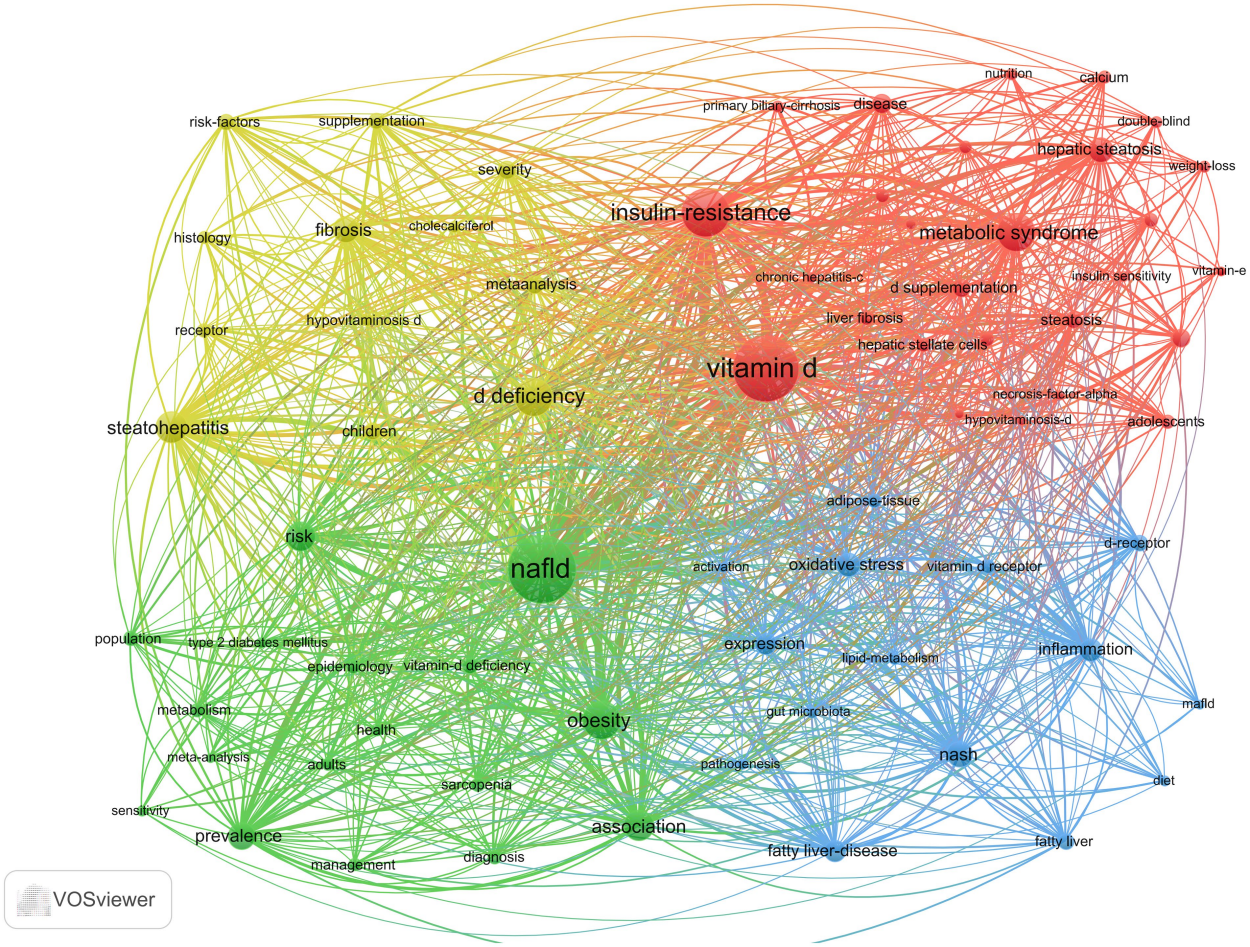
Network of co-occurring keywords.

As illustrated in Figure 10, the keyword co-occurrence network was generated using VOSviewer. In this network, the denser connections between nodes signify a higher frequency of co-occurrence between two keywords. The size of the nodes represents the frequency of each keyword, with larger nodes indicating higher occurrences. Additionally, the colors of the nodes denote different clusters within the network. A total of 1,442 keywords were identified, among which 71 keywords appeared more than nine times. These frequently used keywords were subsequently categorized into four distinct groups, each reflecting one of the primary research directions in the field. The red cluster (cluster #1) is characterized by vitamin D, metabolic syndrome, insulin resistance, and hepatic steatosis. Metabolic syndrome is a group of diseases characterized by obesity, hyperglycemia, dyslipidemia, and hypertension. Insulin resistance represents the core underlying mechanism in the pathogenesis of type 2 diabetes mellitus (T2DM) (15). Vitamin D can also increase insulin sensitivity in body cells (13). It has been shown that liver inflammation, steatosis, and insulin resistance can be ameliorated by activating vitamin D receptors (VDRs) in liver macrophages (16). Furthermore, the VD/VDR axis plays a crucial role in regulating metabolic pathways involved in insulin sensitivity and glucose-insulin homeostasis (17–19). Tingwan Du et al. (20) used a high-fat diet (HFD) model (21) to establish a rat model of NAFLD and successfully demonstrated that VD ameliorates HFD-induced hepatic steatosis.

The green cluster (cluster #2) consists of epidemiological terms such as NAFLD, obesity, prevalence, and epidemiology. MAFLD manifests itself histologically as ≥5% intracellular fat in the hepatocytes, with a higher probability of fibrosis and cancer when ≥30% is present (22). The prevalence of MAFLD in obese individuals or in obese populations with T2DM ranges from 70 to 80% (23), suggesting that obesity and T2DM are important causative factors for MAFLD. MAFLD is reported to be the most common chronic liver disease in the world, and data from four countries, France, Italy, the United Kingdom, and Germany, show that approximately 52 million people suffered from MAFLD in 2016 and that they spend approximately 35 billion euros a year on medical visits (24). Its progress and consequences affect the health and economic systems of Western countries (25).

The blue cluster (cluster #3) focuses on etiology and pathogenesis and is composed primarily of inflammation, NASH, oxidative stress, vitamin D receptors, and pathogenesis. It has been suggested that VD prevents oxidative stress by inhibiting signaling pathways and associated cellular senescence, promoting nuclear factor nuclear red lineage 2-related factor nuclear translocation, reducing Toll-like receptors (TLRs), inhibiting sirtuin, and activating stem cell nuclear factor 4α (HNF 4α) (26–30). For MAFLD, vitamin D receptors (VDRs) are widely expressed in adipose tissue and are involved in the regulation of metabolic disorders (31). Hepatic VDR expression is negatively correlated with the severity of steatosis and lobular inflammation in liver histology (32).

The yellow cluster (cluster #4) consists of vitamin D deficiency, fibrosis, steatohepatitis, histology, severity, and supplementation, focusing on disease manifestation and treatment. MAFLD leads to MASH and cirrhosis, and although the pathogenesis of MAFLD is currently unknown, it has been shown that patients with MAFLD have lower vitamin D levels than healthy controls (33–35). VD has important proliferative and antifibrotic effects on hepatic fibrosis by inhibiting the expression of proliferative and pro-fibrotic markers in hepatic stellate cells, as well as the transitional deposition of extracellular matrix components (36).

## Discussion

### Overall distribution

The literature on VD and MAFLD in the Web of Science database first appeared in 2007. Since then, the annual number of publications has shown a steady increase, indicating growing interest and suggesting that this research area will continue to attract increasing attention in the future.

The citation analysis on the Web of Science search page gives the researcher the frequency of use and citations for each document, which allows for a more objective measure of the frequency of published work by researchers and supports prioritization information for research progress (37, 38). The number of articles published annually reached a maximum of 42 in 2023, which may be caused by the increasing number of studies between VD and MAFLD and possibly by the increase in the number of patients with VD deficiency and MAFLD. As of the date of the search, papers exploring this proposition were continuously updated, indicating that research in this area is still on the rise.

China ranks first in terms of articles, exceeding the second place of the United States by 37 articles, and the number of articles sent by Chinese organizations also accounts for the top three. In the list of authors with the most articles, only one author from China appears on the list, and it is ranked third; this shows that China has some strength in this field, but there is still room for improvement in strengthening the quality of articles. The United States ranked second in terms of the number of articles published, with two of the top 10 publishing organizations coming from the United States, but no authors from the United States appeared in the top 10 authors with the most articles published. Italy ranks third in terms of the number of articles, but the top two authors are from the same institution, indicating that the institution has a high interest in this field and has carried out extensive research with the cooperation of two authors. This highlights China, the United States, and Italy as the principal drivers and foremost contributors to advancing research in this field.

In addition, our results show that Barchetta, Ilaria, is the most influential author in this field, being both the author of the most publications and the most cited author, with four of the top 10 most cited publications coming from Barchetta, Ilaria. Its scientific team found a strong correlation between NAFLD and VD levels before in successive clinical trials between 2011 and 2016 (11). VDR expression negatively correlates with the degree of hepatocyte injury in hepatitis (32). Within patients with T2DM combined with NAFLD, intervention therapy with oral VD supplementation did not improve hepatic steatosis, biochemical indices, endothelial function, and subclinical atherosclerosis of the vasculature (39).

### Hot topics and Frontiers

Based on keyword highlighting and clustering analyses of VD and MAFLD studies, the research hotspots in recent years are as follows:

1. Studies in the etiology, pathogenesis, and epidemiology of the field, such as insulin resistance, hepatic steatosis, obesity, prevalence, inflammation, NASH, oxidative stress, vitamin D receptor; these keywords have been previously discussed and elucidated in the keyword co-occurrence analysis section above.
2. Currently, “acid” is still in an explosive phase. Insulin resistance leads to disturbed catabolism of lipid droplets (LDs) by hepatocytes, which in turn generates large amounts of free fatty acids (FFAs) (40), and the accumulation of excess FFAs in the liver promotes oxidative, inflammatory, fibrotic, and apoptotic signaling pathways (41). While in normal humans, triglyceride accumulation in the liver is a non-toxic, safe form of lipid present in the liver and is indicative of FFAs balance within the hepatocytes (42). 1,25(OH)₂D₃ suppresses LD accumulation in 3 T3-L1 adipocytes by downregulating the expression of genes involved in triglyceride (TG) synthesis while simultaneously upregulating the expression of genes responsible for TG degradation (43). Peroxisome proliferator-activated receptor *α* (PPARα) is a regulator of lipid metabolism and is a favored target for MAFLD therapy. It has been shown that VD increases protein expression of PPARα in the liver and that VD may regulate lipid uptake through the PPARα signaling pathway (20, 44). However, most of the current studies on VD in the regulation of lipid metabolism have focused on the protein level, while relatively few studies have been conducted on the mechanisms at the gene level. Further exploration of the role of VD in regulating the PPARα signaling pathway and other lipid metabolism-related gene expression may provide new research directions for revealing its potential protective mechanisms in MAFLD. In addition, whether VD interacts with other metabolic pathways (e.g., AMPK and SREBP-1c) through the PPARα signaling pathway and the synergistic regulatory mechanisms of these pathways in hepatic lipid homeostasis remains to be further investigated. In the future, the integration of multi-omics approaches, such as transcriptomics and metabolomics, alongside ex vivo and *in vivo* models, holds promise for elucidating the complex network underlying vitamin D’s role in hepatic lipid metabolism. These advanced methodologies could provide a robust conceptual framework for exploring the potential therapeutic applications of VD in the treatment of MAFLD.
3. Osteoporosis is a prevalent systemic bone disorder characterized by reduced bone mass and structural deterioration, which together result in increased bone fragility and a heightened risk of fractures (45). Obesity may be an important factor mediating the relationship between MAFLD and osteoporosis (46). Obesity exacerbates MAFLD and osteoporosis by causing chronic low-grade inflammation in the liver (47), increasing insulin resistance (48), affecting leptin and lipocalin secretion (49, 50), and disrupting intestinal microcirculation (51, 52). Notably, VD may play a protective role in the association of MAFLD with osteoporosis through multiple mechanisms. On the one hand, VD can indirectly attenuate bone damage due to metabolic disorders by increasing insulin sensitivity and improving insulin resistance. On the other hand, as an important regulator of bone metabolism, VD is directly involved in the maintenance of calcium and phosphorus metabolism and promotes the process of bone mineralization. In addition, VD has an immunomodulatory effect, which can alleviate the chronic inflammatory state by reducing the release of pro-inflammatory factors, thus exerting a protective effect against osteoporosis. Current studies on the role of VD in the association between MAFLD and osteoporosis are mostly retrospective, with limited sample size and data quality, and the causal relationship is unclear. Future research should focus on designing well-structured prospective cohort studies and randomized controlled trials to comprehensively evaluate the dual role of VD supplementation in mitigating MAFLD and osteoporosis. These efforts will be critical in advancing our understanding of VD’s therapeutic potential in addressing these interconnected conditions. In addition, the specific mechanisms of VD in intestinal microecological regulation, leptin and lipocalin metabolism, and osteoblast signaling pathways should be explored in depth. By integrating multi-omics analysis and animal model studies, the role network of VD in metabolic liver disease and bone metabolism disorders will be further revealed, providing a scientific basis for VD in the prevention and treatment of related diseases.

### Limitations

This study represents the first comprehensive bibliometric analysis of research hotspots and emerging frontiers in the field of VD and MAFLD over the past 20 years, utilizing both CiteSpace and VOSviewer. By using these advanced analytical tools, this work provides a more accurate and objective foundation for guiding future research in the field. Nonetheless, this study is not without its limitations. First, all data were exclusively extracted from the WOSCC database, which is continuously updated. As a result, literature published after the cutoff date for article retrieval could not be included. Second, given the vast volume of literature in this field and the rapid evolution of emerging topics and research frontiers, there is a possibility that some research hotspots may have been overlooked. Finally, the manual selection process of literature is somewhat subjective.

### Concluding remarks

A total of 359 publications on VD and MAFLD were identified between 2007 and 2024. This study analyzed and summarized the contributions of key authors, institutions, and countries that have significantly advanced research in this field. Simultaneously, we examined the research hotspots and emerging trends within this field. Current studies primarily focus on key targets such as insulin resistance, obesity, osteoporosis, and bile acids. However, the efficacy of VD supplementation in improving MAFLD remains a subject of ongoing debate. The findings of this study are expected to provide valuable insights for advancing future research and development in this area, while also serving as a critical reference for the prevention and treatment of MAFLD.

## Data Availability

The original contributions presented in the study are included in the article/supplementary material, further inquiries can be directed to the corresponding author.
